# Leaky Blood–Brain Barrier and Chronic Pain: The Neuroinflammatory Link

**DOI:** 10.3390/biology15141145

**Published:** 2026-07-14

**Authors:** Mario García-Domínguez

**Affiliations:** Facultad de Educación, Universidad Alfonso X El Sabio (UAX), Avenida de la Universidad, 1, Villanueva de la Cañada, 28691 Madrid, Spain; marigado@uax.es

**Keywords:** chronic pain, blood–brain barrier, central sensitization, neuroinflammation, *neurovascular unit*, glial activation, tight junction disruption

## Abstract

Chronic pain represents a significant clinical challenge and is frequently associated with neuroinflammatory processes. The blood–brain barrier plays a central role in protecting the central nervous system by regulating the passage of molecules and immune cells from the periphery. Emerging evidence indicates that in chronic pain conditions, BBB integrity can be compromised, facilitating the infiltration of pro-inflammatory cytokines, immune cells, and neurotoxic mediators into the CNS. These changes contribute to microglial and astrocyte activation, enhancing central sensitization and the persistence of pain. Animal models and clinical studies suggest that mechanisms including tight junction disruption, oxidative stress, and matrix metalloproteinase release underlie this increased permeability. Understanding BBB modulation in chronic pain not only clarifies disease pathophysiology but also highlights potential therapeutic strategies aimed at preserving or restoring barrier integrity.

## Simple Summary

Chronic pain is closely linked to neuroinflammatory processes and might involve disruption of the blood–brain barrier. The blood–brain barrier normally protects the central nervous system by controlling the movement of molecules and immune cells. In chronic pain conditions, blood–brain barrier integrity is highly reduced, allowing pro-inflammatory cytokines, immune cells, and neurotoxic factors to enter into the CNS. This fact contributes to activation of microglia and astrocytes, promoting central sensitization and chronic pain. Evidence from animal and clinical studies suggests that mechanisms such as tight junction breakdown, oxidative stress, and matrix metalloproteinase activity increase BBB permeability. Understanding these changes may help identify therapeutic strategies to preserve or restore blood–brain barrier function in chronic pain conditions.

## 1. Introduction

Chronic pain is a highly prevalent and disabling condition, affecting approximately 20% of the adult population worldwide [1]. It is defined as pain persisting beyond normal tissue healing time, typically exceeding three to six months, and is now widely recognized not merely as a symptom, but as a disease entity in its own right [2]. Its clinical and societal impact is substantial, being a leading cause of long-term disability and significantly contributing to reduced quality of life, impaired daily functioning, and the development of comorbid psychiatric conditions such as depression and anxiety [3,4]. In addition, chronic pain imposes a major socioeconomic burden through increased healthcare utilization, loss of productivity, and long-term work disability [5,6].

Despite extensive research efforts, current pharmacological strategies remain largely insufficient and are mainly symptom-directed rather than mechanism-based. Commonly employed drug classes include opioids, NSAIDs, anticonvulsants, and antidepressants [7,8,9]. Although these pharmacological agents can confer partial analgesic effects, their effectiveness in chronic pain states is often limited and transient [10]. Additionally, their clinical utility is limited by adverse effects, the development of tolerance and dependence (especially with opioid analgesics) and their inability to ameliorate the underlying neurobiological mechanisms that maintain pain chronification [11].

In contrast to earlier models that viewed chronic pain as a direct consequence of sustained peripheral nociceptive input, contemporary neuroscience increasingly conceptualizes it as a disorder of the CNS [12]. A central feature of chronic pain pathophysiology is central sensitization, defined as an activity-dependent increase in the excitability of nociceptive neurons within the CNS, leading to amplified pain responses to both noxious and non-noxious stimuli (hyperalgesia and allodynia) [13]. This process is intimately associated with maladaptive neuroplasticity, which entails long-term structural and functional reorganization of neural circuits that reinforces, rather than resolves, pain signaling [14].

Within this framework, spinal-level changes (primarily in the spinal dorsal horn) include enhanced excitatory neurotransmission, reduced inhibitory control, and increased responsiveness of nociceptive neurons, facilitating exaggerated transmission of pain signals to higher brain centers [15,16]. Supraspinally, multiple interconnected regions are involved, including the thalamus, anterior cingulate cortex, insula, and prefrontal cortex, all of which contribute to different dimensions of pain processing [17,18]. Central sensitization involves sustained glutamatergic hyperactivity at the synaptic level, largely mediated by NMDAR phosphorylation through Src-family kinases and PKC signaling [19]. This increases Ca^2+^ influx and activates intracellular cascades such as CaMKII, PKA, and MAPK (ERK1/2, p38, and JNK), which converge on transcription factors including CREB and NF-κB [20,21]. The result is long-term gene expression changes that enhance pro-nociceptive signaling (e.g., BDNF, substance P, and dynorphin) and reduce inhibitory GABAergic and glycinergic transmission [22,23]. On the other hand, maladaptive neuroplasticity contributes to chronic pain through reduced KCC2 function, which disrupts Cl^−^ homeostasis and impairs GABA_A_-mediated inhibitory signaling, thus promoting depolarizing rather than inhibitory neuronal responses [24,25].

In parallel, neuroinflammation has emerged as a defining hallmark of chronic pain chronification. Microglial cells, the resident immune cells of the CNS, undergo a transition from a surveillant state to an activated phenotype in response to peripheral nerve injury, sustained nociceptive signaling, or circulating inflammatory mediators [26,27]. Activated microglia upregulate surface receptors such as TLR4, P2X4 and P2X7 purinergic receptors, and CX3CR1, triggering intracellular signaling cascades such as NF-κB activation and assembly of the NLRP3 inflammasome [28,29]. This leads to cleavage and release of IL-1β and IL-18, which amplify synaptic transmission in nociceptive pathways [30]. Astrocytes also experience a reactive transformation characterized by the upregulation of GFAP, the opening of connexin hemichannels, and sustained release of cytokines (IL-6 and TNF-α), chemokines (CCL2), and gliotransmitters (glutamate and ATP), thus maintaining chronic excitation of nociceptive circuits [31].

Within this integrated neuroimmune framework, BBB dysfunction has emerged as a potential contributor to the development and persistence of chronic pain, although its role likely differs among chronic pain disorders. The BBB is a highly specialized barrier interface composed of brain microvascular endothelial cells sealed by tight junction complexes (claudin-5, occludin, and ZO-1), pericytes embedded within the basement membrane, astrocytic endfeet expressing AQP-4, and associated extracellular matrix components [32]. This *neurovascular unit* regulates CNS homeostasis by modulating paracellular permeability, selective transcellular transport (GLUT1, LAT1, and receptor-mediated transcytosis systems), and immune cell trafficking [33].

In chronic pain conditions, BBB integrity is compromised through multiple converging molecular mechanisms. Pro-inflammatory cytokines such as TNF-α and IL-1β induce endothelial activation via NF-κB-dependent transcriptional programs, leading to reduced expression and mislocalization of tight junction proteins [34]. Concurrently, activation of RhoA/ROCK signaling promotes actin cytoskeleton contraction, increasing inter-endothelial gap formation and paracellular leakage [35]. VEGF, usually upregulated in inflammatory and neuropathic states, further destabilizes BBB integrity by increasing endothelial permeability via phosphorylation of tight junction proteins and modulation of vesicular transport pathways [36]. Matrix metalloproteinases (e.g., MMP-2 and MMP-9), released by activated microglia, astrocytes, infiltrating macrophages, and endothelial cells themselves, break down components of the basal lamina (laminin, collagen IV, and fibronectin) and directly cleave tight junction proteins, thereby structurally weakening the BBB [37]. Oxidative and nitrosative stress activation (NADPH oxidases, such as NOX2/NOX4), mitochondrial dysfunction, and iNOS further exacerbates endothelial injury by inducing lipid peroxidation, DNA damage, and mitochondrial depolarization in BBB cells [38]. In parallel, endothelial activation culminates in upregulation of adhesion molecules including ICAM-1, VCAM-1, and selectins (E-selectin and P-selectin), allowing leukocyte tethering, rolling, and transmigration via the endothelial barrier [39]. This process is boosted by chemokine gradients (e.g., CCL2/CCR2 axis), enabling peripheral immune cells (usually monocytes and T lymphocytes) to infiltrate the CNS parenchyma, where they adopt pro-inflammatory phenotypes that further amplify neuroinflammation [40].

On the other hand, BBB disruption permits the uncontrolled entry of circulating proteins and bioactive molecules normally excluded from the CNS, with albumin extravasation being particularly relevant due to its activation of astrocytic TGF-β/SMAD signaling, driving transcriptional reprogramming associated with astrogliosis and persistent synaptic remodeling [41]. In addition, peripheral cytokines, autoantibodies, and fibrinogen can directly modulate neuronal excitability and glial activation, such as microglial activation via CD11b/CD18 integrin signaling [42]. BBB dysfunction also compromises homeostatic transport systems, with altered GLUT1-mediated glucose transport weakening neuronal energy metabolism, reduced efflux capacity via transporters (such as P-glycoprotein) limiting clearance of neurotoxic metabolites and drugs, and dysregulated ion and neurotransmitter precursor transport [43].

Importantly, BBB dysfunction actively contributes to a self-sustaining feed-forward loop in chronic pain, in which peripheral nerve injury or persistent inflammation induces glial activation and cytokine release that disrupt BBB integrity; the resulting barrier breakdown enhances CNS exposure to circulating pro-inflammatory mediators and immune cell infiltration, further driving microglial and astrocytic activation and reinforcing central sensitization even in the absence of ongoing peripheral input. Collectively, evidence from preclinical and clinical studies implicates BBB dysfunction as a key feature across multiple chronic pain conditions, supporting the concept that pain chronification reflects not only neuronal and glial dysregulation, but also dysfunction of the *neurovascular unit*.

This review aims to provide an in-depth mechanistic synthesis of the role of the BBB in chronic pain pathophysiology, with a focus on molecular and cellular mechanisms underlying BBB disruption, its bidirectional interactions with neuroimmune and neuroglial signaling pathways, and its contribution to central sensitization and pain persistence. Finally, emerging therapeutic approaches targeting BBB integrity are decisively evaluated, including modulation of tight junction stability, inhibition of MMP activity, regulation of endothelial inflammation, and restoration of neurovascular coupling, as potential disease-modifying approaches for chronic pain management.

## 2. Blood–Brain Barrier Physiology

### 2.1. Blood–Brain Barrier: Structure and Molecular Integration

The BBB is a highly specialized, evolutionarily conserved multicellular and molecular interface that constitutes the principal regulatory boundary between the systemic circulation and the CNS parenchyma [44]. Rather than acting as a passive anatomical barrier, the BBB operates as a dynamically regulated neurovascular interface that integrates vascular, glial, immune, and extracellular matrix components into a coordinated functional unit that preserves CNS homeostasis and neuronal computational stability [45].

The BBB is composed of BMECs, which display a highly specialized and organotypic endothelial phenotype that is fundamentally distinct from peripheral vascular endothelium [46]. This specialization is established during neurovascular development via reciprocal signaling between neural progenitors and endothelial precursors, leading to a stable transcriptional and epigenetic program characterized by sustained expression of barrier-associated genes and suppression of permeable endothelial signatures [47,48]. A defining feature of BMECs is their pronounced apical-basolateral polarity, which organizes membrane proteins, transporters, and signaling complexes into spatially segregated domains [49]. This polarity is maintained through conserved molecular machinery including PAR, Crumbs, and Scribble complexes, which organize cytoskeletal organization, vesicular trafficking directionality, and junctional assembly [50]. This polarization supports directional transport of nutrients, metabolites, and signaling molecules, while limiting bidirectional diffusion that could otherwise impair ionic and metabolic homeostasis in the CNS [51].

Paracellular impermeability in BMECs is primarily mediated through an exceptionally dense and continuous tight junction network composed of claudin-5 as the core sealing component, supported by occludin and junctional adhesion molecules that form a molecular diffusion barrier with nanometer-scale selectivity [52]. These junctional complexes are anchored to a specialized cytoskeletal scaffold via zonula occludens proteins, enabling intimate coupling between membrane adhesion and actin cytoskeletal dynamics [53]. This organization yields transepithelial electrical resistance (TEER) values among the highest observed in any vascular bed, reflecting a near-complete limitation of passive ionic and solute flux across the BBB interface [54]. On the other hand, transcytotic flux in BMECs is not constitutive but is instead highly inducible and confined to receptor-mediated pathways that permit selective transport of essential macromolecules under stringent regulatory control [55].

**Figure 1:**
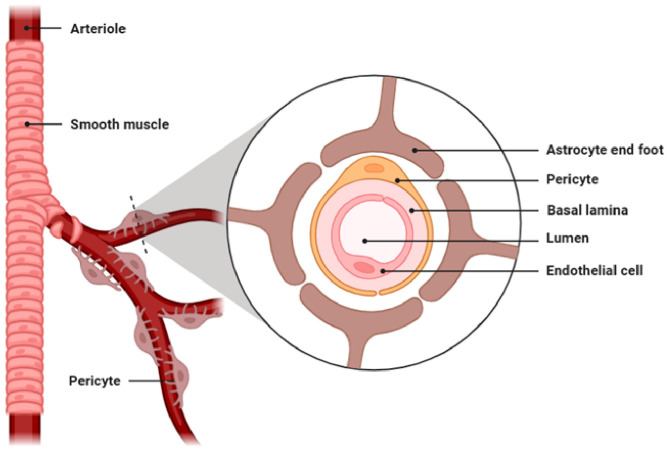
Schematic representation of the *neurovascular unit*, a functional and structural interface in the brain composed of endothelial cells, pericytes, astrocytic endfeet and neurons, which regulate BBB integrity and metabolic coupling between neuronal activity and vascular responses.

Importantly, BMECs do not function in isolation but instead serve as the central component of the *neurovascular unit* (Figure 1), an integrated multicellular system that coordinates vascular, metabolic, and neuronal operations within the CNS [56]. This anatomical unit comprises pericytes embedded within and intimately associated with the vascular basement membrane, where they preserve endothelial stability, vessel maturation, and contractile responses [57,58]. Astrocytic endfeet form a near-continuous envelope around the microvasculature, establishing structural and functional interfaces that contribute to barrier maintenance, ionic buffering, and metabolic optimization of endothelial cells [59]. In addition, astrocytic endfeet participate in barrier maintenance and homeostatic regulation via the polarized expression of AQP-4 and K^+^ channels, as well as the secretion of trophic and barrier-stabilizing factors that maintain endothelial tight junction integrity [60]. Resident immune cells, such as microglia, provide immunosurveillance and modulate vascular and pro-inflammatory responses in the parenchymal milieu [61]. Furthermore, neurons influence cerebrovascular dynamics driven by activity-dependent signaling mechanisms that couple synaptic activity to local blood flow regulation, thus ensuring metabolic supply–demand matching [62]. On the other hand, pericytes play essential roles in providing structural and signaling support by regulating endothelial stability, basement membrane deposition, and vascular contractility mediated by PDGF and TGF-β signaling pathways [63].

At the molecular level, BBB integrity emerges from the coordinated activation of developmental signaling cascades including Wnt/β-catenin, sonic hedgehog, and Notch signaling pathways [64]. Canonical Wnt ligands secreted by neural progenitors and astroglial precursors bind to Frizzled receptors and LRP5/6 co-receptors on endothelial cells, stabilizing cytosolic β-catenin and enabling its nuclear translocation [65]. This transcriptional program upregulates BBB-specific genes such as claudin-5, GLUT1 (SLC2A1), and Mfsd2a while simultaneously repressing genes associated with fenestrated or permeable endothelial phenotypes [66]. Astrocytic sonic hedgehog signaling further reinforces BBB characteristics by suppressing endothelial inflammatory activation via Gli-mediated transcriptional regulation, while Notch signaling contributes to vascular quiescence and tight junction stabilization through Hes/Hey transcriptional effectors [67,68].

Finally, the vascular basement membrane provides an additional structural and signaling scaffold composed of laminins (notably laminin-411 and laminin-511), type IV collagen, nidogens, and heparan sulfate proteoglycans such as perlecan [69,70]. These extracellular matrix components engage endothelial integrins (α1β1 and α6β1) to activate FAK, Src family kinases, and downstream Rho GTPase signaling pathways that orchestrate cytoskeletal tension, endothelial polarity, and junctional assembly [71,72]. Blood-flow-derived shear stress further regulates endothelial transcriptional programs through mechanotransductive complexes consisting of PECAM-1, VE-cadherin, and VEGFR2, which signal through PI3K/Akt and eNOS pathways to coordinate hemodynamic signals with BBB maintenance [73]. Moreover, this matrix not only anchors endothelial cells and pericytes but also regulates mechanotransduction and growth factor availability, thereby modulating barrier phenotype stability [74].

### 2.2. Tight Junctions and Cytoskeletal Control of Paracellular Barrier Function

The defining molecular feature of BBB endothelial cells is the presence of highly specialized and densely organized tight junction complexes that virtually eliminate paracellular diffusion of ions, metabolites, and several hydrophilic solutes [75]. Unlike peripheral endothelial beds, which contain intercellular clefts that facilitate significant paracellular transport, brain microvascular endothelial cells are sealed by continuous tight junctional complexes, resulting in high TEER and extremely low permeability [76]. This phenotype is established during neurovascular development through coordinated signaling between endothelial cells, pericytes, astrocytes, and neural progenitors, and is maintained throughout adulthood by continuous *neurovascular unit* crosstalk [77].

Among the claudin family proteins that form tight junction complexes, claudin-5 is the predominant component and plays a fundamental role in maintaining barrier integrity [78]. Claudin-5 molecules form homophilic interactions across adjacent endothelial membranes, generating continuous anastomosing sealing strands that define the size-selective properties of the BBB [79]. Genetic deletion studies have shown that loss of claudin-5 selectively increases permeability to molecules smaller than approximately 0.8 kDa without causing gross disruption of endothelial architecture, highlighting its role as a molecular sieve [80,81,82]. Other claudins, such as claudin-3 and claudin-12, may contribute to regional heterogeneity of barrier properties, although their precise functions remain incompletely understood [83,84].

Occludin, a tetra-span transmembrane protein enriched at BBB tight junctions, serves both structural and signaling functions. While occludin is not strictly required for barrier formation, its absence significantly affects junctional integrity, signal transduction pathways, and permeability responses in pathological settings [85]. Occludin activity is tightly regulated through phosphorylation-dependent mechanisms involving some kinase pathways, including PKCζ, CK2, Src-family kinases, and MAPKs [86,87,88,89].

Junctional adhesion molecules (JAM-A, JAM-B, and JAM-C), members of the immunoglobulin superfamily, contribute to the reinforcement of endothelial intercellular adhesion and facilitate the coordinated transmigration of leukocytes across the BBB [90]. Beyond their contribution to structural integrity, JAM proteins participate in intracellular signaling pathways like Rap1, integrins, and small Rho GTPases that regulate endothelial polarity and junctional maturation [91]. JAM-A, in particular, contributes to the maintenance of apicobasal polarity and limits immune cell extravasation by modulating interactions with β2 integrins expressed on circulating leukocytes [92].

The intracellular architecture of BBB tight junctions is highly organized by the zonula occludens proteins ZO-1, ZO-2, and ZO-3, which function as multidomain molecular scaffolds linking transmembrane junctional proteins to the actin cytoskeleton [93]. These proteins contain multiple PDZ, SH3, and guanylate kinase-like domains that facilitate the assembly of large multiprotein complexes [94]. By engaging claudins, occludin, JAMs, cortactin, α-catenin, vinculin, and actin-regulatory proteins, ZO proteins organize a dynamic yet mechanically robust junctional platform that enables rapid responses to environmental stimuli [95]. In addition to their mechanical and structural roles, ZO proteins take part in mechanotransduction and transcriptional regulation through interactions with signaling molecules such as ZONAB, thereby coupling barrier integrity to endothelial gene expression programs [96].

Cytoskeletal organization represents a central determinant of BBB permeability and junctional stability. In physiological conditions, endothelial cells primarily display cortical actin ring structures that sustain tight junction architecture and mitigate contractile stress. The balance between barrier stabilization and barrier disruption is largely controlled by members of the Rho family of small GTPases [97]. Rac1 and Cdc42 promote junction assembly, cortical actin polymerization, and endothelial polarity via activation of WAVE and WASP complexes, resulting in Arp2/3-mediated actin branching and stabilization of cell–cell contacts [98,99]. In contrast, RhoA activation stimulates ROCK, leading to phosphorylation of MLCP and increased phosphorylation of MLCs [100]. This process boosts actomyosin contractility, generates centripetal tension, and promotes junctional remodeling [101].

BBB integrity emerges from a dynamic balance between junctional assembly, cytoskeletal tension, intracellular trafficking, and extracellular signaling. The continuous integration of mechanical, metabolic, inflammatory, and neurovascular cues allows endothelial cells to finely tune barrier permeability in real time while maintaining CNS homeostasis. Disruption of these molecular mechanisms is increasingly recognized as a fundamental pathogenic event in numerous neurological disorders, including ischemic stroke, multiple sclerosis, Alzheimer’s disease, Parkinson’s disease, traumatic brain injury, and other neuroinflammatory conditions [102,103].

### 2.3. Suppression of Transcytosis and Highly Selective Transport Systems

Beyond the establishment of restrictive paracellular barriers via tight junction complexes, the integrity of the BBB also critically relies on the suppression of vesicle-mediated transcellular transport [104]. In contrast to peripheral vascular endothelium, BMECs display markedly reduced constitutive transcytosis, a defining feature that contributes substantially to the increased TEER and restrictive permeability of the BBB [54]. This highly specialized phenotype is actively sustained by dedicated molecular programs rather than being a passive outcome of endothelial cell differentiation.

A key regulator of transcytosis suppression is the major facilitator superfamily domain-containing protein 2A (Mfsd2a), a Na^+^-dependent lipid transporter highly enriched in CNS endothelial cells [66]. Mfsd2a is responsible for the uptake of lysophosphatidylcholine (LPC)-bound ω-3 fatty acids, particularly docosahexaenoic acid (DHA), thus modulating the lipid composition and biophysical properties of the endothelial plasma membrane [105]. Through the accumulation of polyunsaturated phospholipid species, Mfsd2a reduces membrane lipid order and disrupts the formation of cholesterol-rich membrane microdomains required for caveolae biogenesis [106]. As a result, caveolin-1 oligomerization and caveolae assembly are markedly inhibited, resulting in a profound reduction in caveolae-mediated endocytosis and transcytosis [107]. Genetic ablation of Mfsd2a results in a dramatic increase in vesicular trafficking across the endothelial cytoplasm, accompanied by enhanced permeability to plasma proteins and several circulating tracers, despite the preservation of ultrastructurally intact tight junctions [108].

While constitutive transcytosis is strongly suppressed, BMECs retain highly selective receptor-mediated transport pathways that ensure the delivery of numerous macromolecules. Clathrin-mediated endocytosis (CME) constitutes the principal mechanism underlying such regulated uptake. Ligand binding induces receptor clustering within phosphatidylinositol-4,5-bisphosphate (PIP_2_)-enriched membrane domains, followed by recruitment of adaptor protein complexes, essentially AP-2, and assembly of the clathrin lattice [109]. Membrane invagination is completed through dynamin-dependent vesicle scission, giving rise to clathrin-coated vesicles that promptly fuse with early endosomes characterized by Rab5 activity [110]. Within these sorting endosomal compartments, cargo fate is determined through a highly regulated network involving endosomal acidification, ubiquitination status, and interactions with sorting nexins and retromer complexes [111].

Metabolic homeostasis within the CNS relies on carrier-mediated transport systems expressed at both luminal and abluminal endothelial membranes. Among these, GLUT1 serves as the main route for cerebral glucose uptake. GLUT1 operates via facilitated diffusion driven concentration gradients, the transporter undergoes conformational transitions between outward-facing and inward-facing states through the alternating-access mechanism [112]. Considering the brain’s exceptional metabolic demands and limited endogenous glycogen stores, GLUT1-mediated glucose transport represents a rate-limiting determinant of neuronal energy metabolism [113]. On the other hand, LAT1 functions as a heterodimer with the glycoprotein CD98, and mediates Na^+^-independent exchange of essential amino acids such as leucine, phenylalanine, tyrosine, tryptophan, and histidine [114]. These substrates are indispensable not only for protein synthesis as well as for the production of several neurotransmitters, including dopamine, norepinephrine, and serotonin [115]. LAT1 operates as an obligatory antiporter, coupling substrate influx to efflux of intracellular amino acids and thereby integrating endothelial transport with intracellular metabolic states [116]. Monocarboxylate transporters, particularly MCT1 and MCT2, allow bidirectional proton-coupled transport of lactate, pyruvate, acetate, and ketone bodies. These transporters play a crucial role in the astrocyte-neuron lactate shuttle hypothesis, whereby glycolytically active astrocytes generate lactate that is subsequently utilized by neurons as an oxidative substrate [117].

In parallel with nutrient uptake systems, the BBB expresses an extensive repertoire of ABC transporters that prevent the accumulation of numerous potentially harmful compounds within the CNS [118]. ABCB1, ABCG2, and MRPs are predominantly localized to the luminal membrane and use ATP hydrolysis to drive substrate extrusion against concentration gradients [119]. These transporters possess broad substrate specificity, recognizing structurally diverse xenobiotics, pharmaceutical agents, environmental toxins, lipid metabolites, pro-inflammatory mediators, and several endogenous signaling molecules [120]. Their activity generates a powerful vectorial efflux system that continuously directs substrates from the endothelial cytoplasm toward the bloodstream. Mechanistically, ATP binding induces nucleotide-binding domain dimerization, driving conformational transitions within transmembrane domains that allow substrate translocation and release [121].

Transport specificity is further enhanced by the pronounced polarization of brain endothelial cells, which establishes distinct luminal (blood-facing) and abluminal (brain-facing) membrane domains. This polarity is maintained by evolutionarily conserved protein complexes like the aPKC complex, the Crumbs–PALS1–PATJ complex, and Scribble-associated polarity networks [122]. These molecular assemblies coordinate cytoskeletal organization, vesicle targeting, membrane protein trafficking, and junctional stability through interactions with Cdc42 and Rac1 [123]. Polarized distribution of transporters, receptors, and efflux pumps ensures directional vectorial transport across the endothelium, allowing nutrients to enter the CNS while simultaneously promoting the elimination of metabolic waste products and xenobiotics [124].

### 2.4. Pericyte and Astrocyte Signaling Networks and Immune Exclusion Mechanisms

Pericytes are mural cells embedded within the endothelial basement membrane that play indispensable roles in BBB development, maintenance, and homeostatic regulation [125]. Beyond their structural association with the microvascular wall, pericytes act as dynamic signaling centers that orchestrate vascular maturation, endothelial specialization, extracellular matrix remodeling, and neuroimmune interactions [126]. The exceptionally high pericyte-to-endothelial cell ratio in the CNS distinguishes cerebral microvessels from most peripheral vascular beds and is considered a key determinant of BBB integrity [127].

Pericytes contribute to BBB integrity through secretion of Ang-1, a vascular stabilizing factor that acts on endothelial Tie2 receptor [128]. Ang-1-induced Tie2 activation leads to Akt phosphorylation and downstream suppression of the transcription factor FOXO1, which is a critical regulator of endothelial permeability and inflammatory gene expression [129]. FOXO1 suppression enhances the membrane localization of junctional proteins such as claudin-5, occludin, and VE-cadherin while simultaneously reducing transcription of genes associated with vascular destabilization [130]. Pericytes also regulate BBB permeability through direct physical interactions with endothelial cells mediated by specialized peg-and-socket contacts, adherens junctions, and gap junctions containing connexins such as Cx43 and Cx45 [131]. These structures allow bidirectional exchange of ions, metabolites, and second messengers, enabling rapid coordination of vascular responses to physiological and pathological stimuli [132]. Emerging evidence further suggests that pericytes contribute to neurovascular coupling by transmitting neuronal activity-dependent signals to the vascular wall, thereby modulating local cerebral blood flow [133].

Astrocytes provide a second major cellular component of the *neurovascular unit* and are involved in BBB maturation, maintenance, and functional regulation [134]. Astrocyte-derived processes terminate in specialized perivascular endfeet that cover over 90% of the cerebral microvascular surface. These endfeet establish an interface between neural tissue and the cerebral circulation, enabling bidirectional communication between neurons, glia, and vascular cells [135]. A molecular feature of astrocytic endfeet is the highly polarized expression of AQP-4, the main water channel of the CNS. AQP-4 is concentrated within orthogonal arrays of particles that are anchored to the perivascular membrane through interactions with the dystrophin-associated glycoprotein complex [136]. This localization drives efficient bidirectional water movement across the glial–vascular interface, contributing to osmotic regulation and CSF-interstitial fluid exchange [137]. AQP-4 is also a crucial component of the glymphatic system, facilitating convective fluid transport involved in metabolic waste clearance, including the removal of amyloid-β and τ proteins from the brain parenchyma [138].

Astrocytic endfeet are further enriched in Kir channels, particularly Kir4.1, which mediate K^+^ spatial buffering. During neuronal activity, extracellular K^+^ concentrations rise locally and must be rapidly cleared to prevent excessive neuronal depolarization and excitotoxicity. Kir4.1 channels facilitate K^+^ uptake by astrocytes, which subsequently redistribute excess ions through interconnected astrocytic syncytia via gap junction networks [139]. This process contributes to the maintenance of extracellular ionic homeostasis and stabilizes neuronal excitability. Beyond their homeostatic functions, astrocytes secrete a broad repertoire of factors, including GDNF, ApoE3, Wnt ligands, retinoic acid, and TGF-β, which regulate endothelial phenotype and sustain BBB-specific gene expression programs [140,141,142].

On the other hand, immune exclusion at the BBB is maintained via multiple overlapping structural and molecular mechanisms that collectively establish a highly restrictive interface between the peripheral immune system and the CNS. Under physiological conditions, BMECs exhibit an immunologically quiescent phenotype characterized by minimal expression of several leukocyte adhesion molecules such as ICAM-1, VCAM-1, P-selectin, and E-selectin. The low abundance of these molecules prevents leukocyte tethering, rolling, and firm adhesion along the vascular endothelium [143]. During neuroinflammatory conditions, activation of PRRs and/or cytokine receptors, triggers profound endothelial reprogramming [144]. Several pro-inflammatory mediators (e.g., TNF-α, IL-1β, IFN-γ, and IL-17) strongly activate NF-κB, JAK/STAT, and MAPK signaling pathways. These transcriptional programs evoke the upregulation of adhesion molecules including ICAM-1, VCAM-1, E-selectin, and P-selectin, as well as chemokines like CCL2, CCL5, CXCL10, and CXCL12 [145,146,147]. The resulting chemotactic milieu facilitates the recruitment of circulating leukocytes to sites of CNS inflammation.

## 3. BBB Dysfunction in Chronic Pain

### 3.1. Evidence of BBB Disruption in Chronic Pain Conditions

Chronic pain is recognized as a state of sustained neurovascular and neuroimmune dysregulation in which BBB integrity is progressively compromised through coordinated molecular remodeling of the *neurovascular unit* [148]. In various preclinical neuropathic pain models, BBB disruption is not merely a permeability phenomenon but reflects transcriptional reprogramming of endothelial cells, pericytes, and astrocytes (Table 1) [149,150,151,152,153,154]. After nerve injury, endothelial cells demonstrated rapid activation of stress-response kinases, including p38 MAPK and JNK, which converge on transcription factors including NF-κB, AP-1, and STAT3 [155,156]. This leads to downregulation of BBB-enriched genes such as *CLDN5*, *OCLN*, and *TJP1*, mediated in part by epigenetic modifications including histone H3K27 acetylation changes and DNA methylation shifts at promoter regions [157,158].

Simultaneously, there is suppression of Wnt/β-catenin signaling, a pathway essential for BBB maintenance. In several chronic pain states, reduced astrocytic Wnt7a/Wnt7b release leads to decreased stabilization of endothelial β-catenin, preventing its nuclear translocation and transcription of BBB-maintaining genes such as claudin-5 and GLUT1 [159]. In parallel, Notch signaling becomes dysregulated, with altered DLL4/Notch1 interactions contributing to endothelial activation and loss of quiescence [160]. These transcriptional changes are linked to structural disorganization of tight junction complexes and increased paracellular diffusion [161].

### 3.2. Neurovascular Unit Failure and Mechanisms of Increased BBB Permeability

Under physiological conditions, endothelial barrier stability is robustly sustained by a regulated signaling network focused on Wnt/β-catenin, angiopoietin-1/Tie2, and S1PR1. S1PR1 activation promotes Rac1-mediated cytoskeletal stabilization and cortical actin formation, suppressing endothelial contraction [162]. Angiopoietin-1 binds to Tie2 and activates PI3K/Akt signaling, thus preventing FOXO1 from translocating to the nucleus and maintaining junctional integrity [163]. In chronic pain, this equilibrium is disrupted by a shift toward angiopoietin-2 dominance, which competitively inhibits Tie2 signaling, leading to endothelial destabilization and augmented responsiveness to pro-inflammatory cytokines [164].

TNF-α and IL-1β signaling induce strong activation of RhoA via GEF-H1 and p115-RhoGEF, promoting downstream ROCK1/ROCK2 activation [165,166]. ROCK phosphorylates MLC via inhibition of MLCP, resulting in actomyosin contraction and mechanical pulling apart of tight junctions [167]. This cytoskeletal tension disrupts claudin-5/occludin anchoring to ZO-1 and the actin cytoskeleton [168]. Concomitantly, VEGF-A signaling via VEGFR2 activates Src kinase, which phosphorylates occludin on numerous Tyr residues, triggering clathrin-mediated endocytosis of tight junction complexes. This process is further amplified by caveolin-1-dependent transcytosis, which is strongly upregulated in inflammatory states via NF-κB-dependent transcriptional control [169]. Pericyte loss or detachment, mediated by PDGF-B/PDGFR-β signaling disruption, reduces secretion of angiopoietin-1 and TGF-β, removing critical stabilizing cues [170]. Astrocytes, under chronic inflammatory stimulation, undergo A1 reactive transformation driven by IL-1α, TNF-α, and C1q, leading to loss of BBB-supportive functions such as lactate shuttle regulation, K^+^ buffering via Kir4.1 channels, and AQP-4 polarization at end-feet [171,172].

### 3.3. Entry and Amplification of Peripheral Immune and Neurotoxic Signals

Once BBB integrity is compromised, a molecular influx of cytokines, immune cells, and DAMPs initiates a self-sustaining neuroinflammatory cascade [173]. TNF-α binding to TNFR1 recruits TRADD, RIPK1, and TRAF2, leading to activation of the IKK complex (IKKα/IKKβ/NEMO), which phosphorylates IκBα and releases NF-κB for nuclear translocation [174,175]. This results in transcription of IL-1β, IL-6, CCL2, and COX-2, amplifying prostaglandin synthesis through mPGES-1 coupling [176]. IL-1β signaling through IL-1R1 activates MyD88, IRAK4, and TRAF6, leading to NF-κB and MAPK activation, reinforcing pro-inflammatory gene expression [177].

Microglia respond to this milieu by adopting a disease-associated, pro-inflammatory phenotype characterized by activation of TLR4 and CD14 and engagement of downstream MyD88- and TRIF-dependent signaling pathways [178,179]. This triggers assembly of the NLRP3 inflammasome, including ASC oligomerization and activation of caspase-1, which subsequently processes pro-IL-1β into its mature and active form [180]. On the other hand, ATP released from damaged neurons activates P2X7 receptors on microglia, evoking K^+^ efflux, a key trigger for NLRP3 activation [181].

Immune cell infiltration is mediated by numerous adhesion cascade steps involving selectin-mediated rolling (E-selectin and P-selectin), integrin activation (LFA-1 and VLA-4), and firm adhesion through ICAM-1 and VCAM-1 engagement [182,183]. Endothelial chemokine presentation of CCL2, CX3CL1, and CCL5 drives directional migration gradients [184]. Transmigration occurs via CD31, JAM-A, and CD99-mediated junctional opening, combined with localized degradation of basement membrane components by MMP-9 secreted by both leukocytes and activated endothelial cells [185,186,187,188].

Finally, several neurotoxic mediators (e.g., glutamate) accumulate due to impaired EAAT1/EAAT2 astrocytic uptake, itself downregulated by TNF-α-mediated NF-κB activation [189]. Increased extracellular glutamate promotes NMDA receptor overactivation, leading to Ca^2+^ influx through NR2B-containing receptors, activation of calpain, NO synthase, and mitochondrial permeability transition pore opening [190,191]. This fact results in ROS amplification, cytochrome c release, and synaptic remodeling favoring long-term potentiation of nociceptive pathways [192].

### 3.4. Molecular Mechanisms Underlying BBB Breakdown

The structural collapse of the BBB in chronic pain is driven by a convergence of pro-inflammatory signaling, oxidative stress, and extracellular matrix degradation, which collectively dismantle endothelial junctional integrity [150]. Tight junction disruption is primarily mediated by TNF-α and IL-1β signaling through TNFR1 and IL-1R1 receptors, respectively, activating downstream NF-κB, JNK, and p38 MAPK pathways [193,194]. These cascades promote transcriptional repression of claudin-5 and occludin, while simultaneously inducing phosphorylation of occludin and ZO-1 via PKCζ and Src-family kinases [195]. Additionally, TNF-α-induced NF-κB activation blocks CLDN5 transcription via recruitment of HDAC1/2 to several promoter regions, resulting in chromatin condensation and gene silencing [196]. These modifications result in dissociation of tight junction complexes from the actin cytoskeleton and their subsequent internalization and degradation [197].

Oxidative stress represents a central amplifying mechanism in BBB dysfunction. Activation of NADPH oxidases, mainly NOX2 in microglia and NOX4 in endothelial cells, generates excessive superoxide anions that react with NO produced by uncoupled eNOS due to BH4 depletion, forming peroxynitrite (ONOO^−^), a highly reactive nitrogen species [198,199]. Peroxynitrite promotes lipid peroxidation of endothelial membranes, oxidation and nitration of tight junction proteins such as claudin-5 and ZO-1, and disruption of protein–protein interactions through tyrosine residue modification, thus compromising barrier integrity [200]. Concurrently, mitochondrial dysfunction (characterized by impaired electron transport chain activity at complexes I and III) enhances ROS production via electron leakage, perpetuating redox imbalance [201]. This oxidative environment reinforces endothelial injury and promotes sustained barrier disruption while engaging redox-sensitive transcription factors such as NRF2 [202]; however, chronic activation leads to compensatory exhaustion of antioxidant defenses, including SOD and glutathione peroxidase systems, thus establishing a self-perpetuating cycle of oxidative stress and inflammatory signaling that drives progressive BBB breakdown [203]. Selective NOX2 inhibition (e.g., with apocynin) dampen neuroinflammatory processes, reduce leukocyte recruitment, and preserve tight junction integrity during the early stages of BBB injury [204]. On the other hand, selective NOX4 inhibition (e.g., with GKT136901) offers greater protection against chronic vascular damage, endothelial apoptosis, and fibrosis-like changes associated with neurological disorders [205].

Matrix metalloproteinases, particularly MMP-2 and MMP-9, act as terminal effectors of structural degradation within the BBB [206]. Their expression is induced by NF-κB and AP-1 activation and post-translationally initiated by removal of their pro-domain by plasmin and ROS-mediated cleavage [207]. Once activated, MMP-2 and MMP-9 degrade key components of the vascular basement membrane, including collagen IV, laminin, and fibronectin, leading to destabilization of the endothelial scaffold [208]. Importantly, the activity of MMPs is normally regulated by TIMPs, particularly TIMP-1 and TIMP-2; however, in some chronic pain states, this balance is shifted toward proteolysis, liberating bioactive fragments such as laminin peptides that further activate integrin signaling in immune cells, reinforcing leukocyte recruitment [209,210].

### 3.5. Integrated Pathophysiological Model of BBB Dysfunction in Chronic Pain

**Figure 2:**
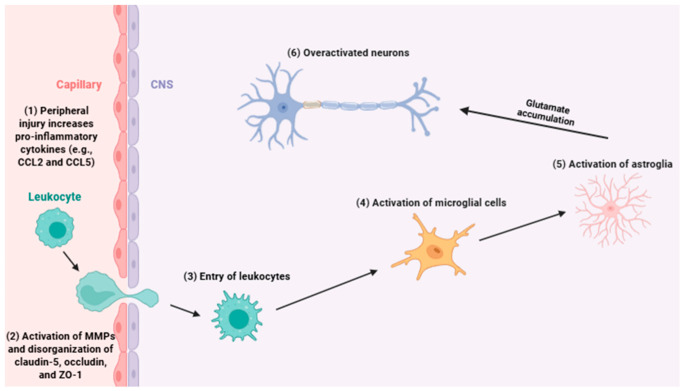
The figure illustrates the progressive loss of BBB integrity following peripheral injury. The scheme is based primarily on evidence from chronic pain studies and summarizes the main mechanisms described under these pathological states. In normal conditions, tight junctions between endothelial cells, supported by pericytes and astrocytic end-feet, maintain barrier function. Upon injury or inflammation, endothelial activation and pro-inflammatory signaling lead to tight junction breakdown, increased transcytosis, and basement membrane alterations. This results in a significant enhancement BBB permeability and the entry of several immune cells into the brain, contributing to neuroinflammation and neural damage. Abbreviations: CCL2 (C-C motif chemokine ligand 2), CCL5 (C-C motif chemokine ligand 5), and ZO-1 (zonula occludens 1).

BBB dysfunction in chronic pain should be conceptualized not as a static breakdown of structural integrity, but as a dynamically regulated and self-amplifying neuroimmune circuit embedded as part of a wider peripheral–central inflammatory axis (Figure 2) [211]. In this framework, persistent peripheral nociceptive input (originating from injured or sensitized tissues) drives systemic immune activation characterized by increased circulating levels of pro-inflammatory cytokines such as TNF-α, IL-1β, and IL-6, alongside various chemokines (CCL2 and CCL5) [212]. These mediators act directly on cerebral microvascular endothelial cells, triggering intracellular signaling cascades dominated by NF-κB and MAPK pathways [213]. Activation of NF-κB promotes transcription of adhesion molecules, proinflammatory enzymes, and additional cytokines, thereby establishing an autocrine pro-inflammatory loop within the *neurovascular unit*.

Concomitantly, cytokine-driven signaling suppresses canonical Wnt/β-catenin pathways, which are essential for maintaining BBB integrity. Inhibition occurs through upregulation of GSK3β activity and enhanced β-catenin phosphorylation and proteasomal degradation, leading to reduced transcription of tight junction components [214]. This results in decreased expression and disorganization of claudin-5, occludin, and ZO-1, weakening paracellular sealing [215]. Pharmacological inhibition of GSK3β has emerged as a promising strategy to normalize Wnt/β-catenin signaling and preserve BBB integrity. In several experimental models of chronic pain, GSK3β inhibitors (e.g., TDZD-8), have been shown to stabilize β-catenin, enhance the expression of tight junction proteins, attenuate neuroinflammation, and reduce mechanical allodynia and thermal hyperalgesia [216,217,218]. In parallel, endothelial cytoskeletal remodeling is driven by activation of the RhoA/ROCK pathway, promoting actomyosin contractility, stress fiber formation, and junctional tension that further increases paracellular permeability [219]. Additional contributions arise from VEGF signaling and MMPs (e.g., MMP-2 and MMP-9), which cleave basement membrane components and cleave tight junction proteins, accentuating barrier disruption [220]. The dysfunction of supporting cells within the *neurovascular unit* (e.g., pericyte detachment and astrocytic endfeet dysregulation) further destabilizes BBB homeostasis [221].

Once BBB integrity is compromised, some peripheral immune cells (monocytes/macrophages and T lymphocytes) gain increased access to the CNS parenchyma via enhanced diapedesis, facilitated by chemokine gradients (notably CCL2-CCR2 signaling) and adhesion molecule upregulation [222,223]. Within the CNS, these infiltrating cells interact with resident microglia, which are primed via pattern recognition receptors (such as TLR4) and induce downstream NF-κB and NLRP3 inflammasome signaling [224]. Inflammasome assembly leads to caspase-1 activation and subsequent maturation and release of IL-1β and IL-18, reinforcing a pro-inflammatory feed-forward loop [225]. Microglia also form ROS via NOX2 activation, further potentiating oxidative stress and redox-sensitive pro-inflammatory signaling [226].

Astrocytes respond to this milieu by adopting a reactive phenotype characterized by altered glutamate homeostasis, like downregulation of excitatory amino acid transporters (EAAT1/EAAT2), leading to extracellular glutamate accumulation [227]. This fact implies overactivation of NMDA and AMPA receptors on neurons, enhancing Ca^2+^ influx and excitatory synaptic drive [228]. Concurrently, inhibitory GABAergic tone is reduced through interneuron dysfunction and altered Cl^−^ homeostasis, modifying the excitation–inhibition balance toward hyperexcitability [229]. These changes result in central sensitization, characterized by synaptic potentiation, lowered activation thresholds, and expanded receptive fields in nociceptive circuits [230].

Finally, this sensitized state participates in a feedback loop with central and peripheral compartments. Enhanced spinal and supraspinal output can facilitate neurogenic inflammation in peripheral tissues via antidromic signaling and release of substance P and CGRP, sustaining peripheral immune activation [231]. Thus, a self-reinforcing loop is established in which peripheral inflammation disrupts BBB integrity, central neuroinflammation amplifies neuronal excitability, and altered central output perpetuates peripheral immune signaling. This integrated neuroimmune-vascular circuit provides a mechanistic framework for the persistence and progression of chronic pain states.

## 4. Therapies Targeting BBB Dysfunction in Chronic Pain

### 4.1. Preclinical Results

Firstly, statins in preclinical studies consistently show protective effects on both the BBB and BSCB in experimental neuropathic pain models. In rodent models of neuropathy, systemic administration of rosuvastatin or simvastatin prevents and reverses mechanical allodynia and thermal hyperalgesia (Table 2) [232,233,234,235]. These effects are accompanied by marked reductions in spinal cord microglial and astrocytic activation, as well as suppression of pro-inflammatory mediators, such as IL-1β [233,235]. Importantly, statins reduce BBB-related inflammatory signaling and endothelial dysfunction, likely via inhibition of oxidative stress and MMPs, which are crucial drivers of barrier disruption [236].

Secondly, minocycline is one of the most extensively studied preclinical agents linking BBB dysfunction, neuroinflammation, and chronic pain. In numerous rodent neuropathic pain models, minocycline reduces mechanical and thermal hypersensitivity when administered early after injury (Table 2) [237,238,239,240,241,242,243]. Mechanistically, it inhibits microglial activation through suppression of p38 MAPK signaling and TLR4 pathways, thereby reducing downstream cytokine release and excitatory neurotransmission in the dorsal horn. Although minocycline is not a direct BBB-targeted drug, experimental evidence indicates that it stabilizes neurovascular function by limiting MMP expression and reducing glial-mediated propagation of inflammatory signals across the BBB [250]. These effects are particularly relevant because microglial activation is a consequence and amplifier of barrier disruption in chronic pain states. On the other hand, in several rodent pain models, pharmacological inhibition of ROCK reduces mechanical allodynia and restores BBB integrity (Table 2) [244,245,246]. These effects are linked to decreased endothelial contraction, reduced leukocyte infiltration, and blockade of pro-inflammatory cascades within the spinal cord. These findings support ROCK as a mechanistic regulator of BBB integrity in pain states, with consistent translational potential. Additionally, COX inhibition provides mechanistic evidence linking inflammatory signaling to BBB disruption by limiting downstream pro-inflammatory cascades and cytokine amplification (Table 2) [247,248,249].

Finally, the robust preclinical evidence demonstrating that modulation of microglial activation and pro-inflammatory pathways preserves BBB integrity and attenuates pain hypersensitivity has prompted their evaluation in clinical settings. Consequently, several clinical trials have investigated whether these mechanisms translate into meaningful therapeutic benefits in patients with chronic pain. The following section summarizes the available clinical evidence on interventions aimed at modulating BBB integrity in patients with chronic pain.

### 4.2. Evidence from Clinical Trials

#### 4.2.1. Indirect Effects on the Blood–Brain Barrier

Among clinically available BBB-related therapies, TNF-α antagonists have generated some of the strongest translational evidence. The study conducted by Genevay et al. demonstrated substantial improvements in pain and disability following s.c. etanercept (mAb against TNF-α) administration in patients with severe sciatica, providing one of the earliest clinical demonstrations that cytokine blockade might influence pain pathways associated with neurovascular dysfunction [251]. Although no direct assessment of BBB integrity was performed, the biological plausibility is compelling because TNF-α is a well-established regulator of BBB permeability. Subsequent trials will be crucial to establish if treatments against TNF-α are critical to restore BBB integrity in chronic pain conditions.

Minocycline is one of the best-characterized pharmacological inhibitors of microglial activation and has attracted considerable interest as a therapy targeting neuroinflammatory mechanisms downstream of BBB breakdown [252]. In a randomized, double-blind, placebo-controlled clinical trial, Vanelderen et al. evaluated minocycline in patients with lumbar radicular neuropathic pain and observed significant reductions in pain intensity compared with placebo [253]. However, the magnitude of benefit was relatively modest and of uncertain clinical relevance. Despite these limitations, this study remains one of the few original clinical trials that directly test a drug developed from preclinical evidence linking neuroinflammation, BBB dysfunction, and chronic pain.

Finally, low-dose naltrexone (LDN) has been proposed as a contributor of neuroinflammation through antagonism of TLR4 signaling in activated microglia. Whereas it does not directly target BBB integrity, its mechanism analyzes downstream consequences of BBB dysfunction, particularly glial activation [254]. The most foundational original study was conducted by Younger et al. (2013), who reported significant reductions in fibromyalgia pain severity in a randomized, double-blind, placebo-controlled trial involving women with fibromyalgia [255]. More recently, larger placebo-controlled studies have produced more equivocal findings, highlighting ongoing uncertainty regarding the magnitude of its clinical efficacy [256,257]. Despite this, LDN remains one of the most frequently discussed translational therapies linking neuroimmune modulation and chronic pain.

#### 4.2.2. Direct Effects on the Blood–Brain Barrier

Focused ultrasound (FUS) represents a different therapeutic strategy because directly modifies BBB permeability rather than pharmacologically restoring BBB integrity [258]. In conjunction with i.v. administered microbubbles, FUS can transiently open the BBB, facilitating targeted delivery of biologics, antibodies, gene therapies, or analgesic compounds to pain-processing brain regions [259,260,261]. Clinical trials in humans have demonstrated the applicability and safety of BBB opening in neurological disorders like AD and brain tumors [262,263]. However, applications in chronic pain remain in early translational development, and no large randomized clinical studies have yet established efficacy. Thus, FUS serves more as a drug-delivery platform than as a pain therapy.

## 5. Conclusions

BBB dysfunction has emerged as a mechanistically relevant component in the pathophysiology of neuroinflammation and chronic pain. Rather than functioning solely as a passive anatomical interface, the BBB should be understood as a dynamic and highly regulated *neurovascular unit* that actively contributes to CNS homeostasis. Increasing evidence indicates that BBB impairment is not merely a downstream consequence of inflammatory processes, but a driving force that sustains and amplifies neuroimmune activation, thereby promoting the transition from acute to chronic pain states.

In this framework, BBB disruption represents a critical nexus between peripheral immune signals and central neuroinflammatory pathways. Increased permeability facilitates the entry of circulating immune cells and numerous pro-inflammatory mediators into the CNS, where they interact with resident glial cells and neurons. This interaction reinforces a self-perpetuating feed-forward loop in which inflammation induces further BBB breakdown, and barrier dysfunction exacerbates neuroinflammation. Over the course of time, this process contributes to central sensitization and maladaptive neuroplastic changes that underlie persistent pain perception. Importantly, this conceptual change reframes chronic pain disorders as systemic neuroimmune-vascular conditions rather than purely neuronal hyperexcitability syndromes. Within this revised paradigm, the BBB is no longer considered a bystander or epiphenomenon, but an active pathogenic driver that shapes disease progression. Its dysfunction influences multiple levels of CNS organization, like immune cell trafficking, glial reactivity, synaptic transmission, and network-level plasticity. On the other hand, emerging evidence suggests that BBB function and neuroimmune responses differ between males and females. Sex hormones modulate BBB permeability, endothelial function, and glial activation, which may contribute to the higher prevalence and distinct clinical manifestations of several chronic pain disorders [264,265]. These observations reinforce the importance of considering sex as a biological variable when investigating BBB dysfunction and developing targeted therapeutic strategies.

Therapeutic strategies aimed at restoring BBB integrity might therefore exert disease-modifying effects by interfering the reciprocal interactions between vascular dysfunction and neuroinflammation. Such interventions (e.g., ibuprofen and/or corticosteroids) have the potential to reduce leukocyte infiltration, normalize glial activity, and restore neuronal homeostasis. As a result, the BBB has emerged as a promising therapeutic target for some chronic pain conditions characterized by persistent neuroimmune activation, including neuropathic pain, fibromyalgia, migraine, MS-associated pain, and CRPS.

Moreover, normalization of BBB function is likely to influence glial biology, particularly microglial and astrocytic activation states. Given the role of glial cells in maintaining neuroinflammatory environments, restoring their homeostatic phenotype may reverse the central sensitization process by rebalancing excitatory and inhibitory neurotransmission and reducing hyperexcitability within pain-processing circuits. Conversely, BBB restoration may also assist the recovery of physiological network dynamics by limiting aberrant neuroimmune signaling that alters synaptic integration and plasticity, which is critical in chronic pain conditions characterized by persistent sensitization and CNS circuit reorganization. The findings summarized in this review suggest that BBB dysfunction may contribute to the maintenance of chronic pain and represents a promising therapeutic target. However, more direct clinical evidence is still needed to establish the causal relationship between BBB restoration and pain resolution, as well as to validate BBB-targeted interventions in patients.

Finally, this BBB-centered framework highlights the necessity of an interdisciplinary research agenda integrating vascular biology, immunology, neuroscience, and pharmacology. Future studies should prioritize the identification of reproducible biomarkers of BBB dysfunction, the characterization of patient subgroups most likely to respond favorably from barrier-targeted therapies, including sex-specific differences in BBB biology and neuroimmune responses, and the development of original pharmacological and biological interventions capable of safely and effectively restoring BBB integrity.

## Figures and Tables

**Table 1:** BBB alterations across experimental pain models. Abbreviations: i.pl. (intraplantar), BBB (blood–brain barrier), ZO-1 (zonula occludens-1), CFA (Complete Freund’s Adjuvant), [^14^C] (carbon-14), CCI (chronic constriction injury), EBA (Evans blue albumin), mRNA (messenger RNA), STZ (streptozotocin), ^131^I-BSA (iodine-131-labeled bovine serum albumin), iBEC (induced blood–brain barrier endothelial cell), GLUT1 (glucose transporter 1), GFAP (glial fibrillary acidic protein), TNC (trigeminal nucleus caudalis), BCP (bone cancer pain), IL-1β (interleukin-1 beta), TNF-α (tumor necrosis factor alpha), and MMP-9 (matrix metalloproteinase-9).

Pain Type	Pain Model	Assessment of BBB Permeability	CNS Region Analyzed	Key Findings	References
Inflammatory	I.pl. λ-carrageenan	Brain perfusion with [^14^C]sucrose	Brain	BBB permeability exhibited a biphasic increase accompanied by occludin downregulation and a transient upregulation of ZO-1 expression.	[149]
	I.pl. CFA	Brain perfusion with [^14^C]sucrose	Brain	After CFA-induced inflammation, more sucrose entered the brain, indicating a leakier BBB. This change was linked to altered tight junction proteins: occludin decreased, while claudin-3 and claudin-5 increased, with no change in supporting proteins.	[150]
Neuropathic	CCI	Immersion with EBA Sodium fluorescein	Spinal cord	Spinal cord pericyte loss is associated with disruption of BBB integrity, indicating a link between vascular support cell degeneration and barrier breakdown. This loss correlates with reduced mRNA expression of ZO-1, occludin, claudin-1, and claudin-5.	[151]
	STZ	Incubation with ^131^I-BSA	In vitro model (iBECs)	STZ directly damages BBB cells and astrocytes in vitro. In brain endothelial cells, STZ reduced barrier integrity, disrupted tight junctions, decreased GLUT1 expression, and reduced cell numbers, indicating BBB breakdown. In astrocytes, it lowered GFAP levels and cell viability, suggesting toxicity and reduced proliferation.	[152]
	Migraine	Sodium fluorescein	Trigeminal nucleus caudalis	BBB permeability increased in the TNC during both episodic and chronic stages, while glial activation appeared only in the chronic stage. These effects were region-specific and accompanied by trigeminal hypersensitivity. Overall, these findings suggest that BBB modulation in the TNC contributes to migraine pathophysiology.	[153]
Cancer pain	BCP	Evans blue dye	Spinal cord	BBB disruption is associated with activation of astrocytes and microglia, downregulation of ZO-1 and claudin-5, and elevated IL-1β, TNF-α, and MMP-9 expression.	[154]

**Table 2:** Overview of pharmacological interventions in experimental inflammatory, neuropathic, and cancer pain models, highlighting glial and neuroinflammatory mechanisms and their CNS actions in relation to BBB permeability and drug delivery. Abbreviations: i.t. (intrathecal), i.cv. (intracerebroventricular), PSNL (partial sciatic nerve ligation), IL-1β (interleukin-1 beta), Iba-1 (ionized calcium-binding adaptor molecule 1), GFAP (glial fibrillary acidic protein), PTX (paclitaxel), TBARS (thiobarbituric acid reactive substances), GSH (reduced glutathione), GSSG (oxidized glutathione), PHN (postherpetic neuralgia), SCI (spinal cord injury), IL-6 (interleukin-6), JAK2 (Janus kinase 2), STAT3 (signal transducer and activator of transcription 3), IoN (infraorbital nerve injury), CFA (complete Freund’s adjuvant), PNI (peripheral nerve injury), CCI (chronic constriction injury), BCP (bone cancer pain), OX-42 (CD11b microglial marker), p38 MAPK (p38 mitogen-activated protein kinase), PSD95 (postsynaptic density protein 95), NF-κB (nuclear factor kappa-light-chain-enhancer of activated B cells), Y27632 (Rho-associated protein kinase inhibitor), DLC2 (deleted in liver cancer 2), RhoA (Ras homolog family member A), ROCK (Rho-associated coiled-coil containing protein kinase), ROCK1/2 (Rho-associated protein kinase isoforms 1 and 2), MARCKS (myristoylated alanine-rich C-kinase substrate), CNS (central nervous system), PGE2 (prostaglandin E2), NS398 (selective COX-2 inhibitor), λ-carrageenan (lambda carrageenan), SNL (spinal nerve ligation), COX-2 (cyclooxygenase-2), COX-1 (cyclooxygenase-1).

Pharmacologic Class	Drugs	Pain Type	Key Findings	References
Statins	Simvastatin	Inflammatory (formalin test)	Simvastatin administered i.t. produced dose-dependent antinociception in the formalin test, selectively reducing the second phase of nociception without affecting the acute phase. No effect was observed following i.cv. administration.	[232]
	Rosuvastatin Simvastatin	Neuropathic (PSNL)	Rosuvastatin and simvastatin prevented and reversed neuropathic pain behaviors. These effects were associated with reduced IL-1β expression and decreased spinal microglial and astrocyte activation (Iba-1 and GFAP).	[233]
	Rosuvastatin (+ duloxetine)	Neuropathic (Paclitaxel)	Rosuvastatin and duloxetine reduced mechanical allodynia and thermal hyperalgesia, with isobolographic analysis revealing a synergistic interaction when combined at reduced doses. The combination also decreased spinal astrocyte activation (GFAP expression), without affecting motor performance.	[234]
	Rosuvastatin	Neuropathic (PTX and PSNL)	Treatment with rosuvastatin reduced mechanical and thermal hypersensitivity while decreasing spinal proinflammatory markers (IL-1β and TBARS) and restoring antioxidant balance (GSH/GSSG ratio). These effects were associated with attenuation of neuroinflammatory and oxidative stress responses.	[235]
Tetracyclines	Minocycline	Neuropathic (PHN)	Minocycline has shown antiallodynic effects and can reduce morphine tolerance in PHN model. Therefore, combining morphine with minocycline may enhance analgesic efficacy while limiting opioid tolerance by targeting neuroinflammatory mechanisms.	[237]
		Neuropathic (SCI)	After SCI, activation of microglia and astrocytes contributes to chronic neuropathic pain. Minocycline attenuated IL-6/JAK2/STAT3 activation and restored nociceptive thresholds, suggesting that modulation of this pathway may be a therapeutic strategy for post-SCI neuropathic pain.	[238]
		Neuropathic (IoN)	Trigeminal nerve injury induces microglial activation and subsequent astrocyte activation in the trigeminal spinal subnucleus caudalis, contributing to orofacial neuropathic pain. Minocycline attenuates microglial activation, reduces glial markers and neuronal activation, and alleviates mechanical hypersensitivity.	[239]
		Inflammatory (CFA) Neuropathic (PNI)	In a rat PNI model, neuropathic pain was associated with marked microglial activation and enhanced ATP-dependent P2X signaling, whereas inflammatory pain (CFA) showed weaker astrocytic involvement. Neuronal hyperexcitability was reduced in neuropathic pain by microglial inhibition (minocycline), p38 MAPK blockade, and P2X receptor antagonism, while astrocytic inhibition, gap junction blockade, and JNK inhibition were more effective in inflammatory pain.	[240]
		Inflammatory (formalin) Neuropathic (CCI)	Microglia and astroglia contribute differently to inflammatory and neuropathic pain in a sex-dependent manner. Inhibition of microglial signaling (minocycline) reduced pain predominantly in males, whereas astroglial inhibition (L-α-aminoadipate) attenuated neuropathic pain in both sexes.	[241]
		Cancer pain (BCP)	Microglial activation and BDNF release contribute to BCP. I.t. administration of minocycline prevented mechanical hypersensitivity and reduced microglial activation (OX-42), p38-MAPK phosphorylation, and spinal BDNF expression.	[242]
			Minocycline reduces BCP in rats by modulating glial and inflammatory signaling. In a Walker 256 tumor model, i.t. and systemic minocycline reversed mechanical allodynia and decreased spinal astrocyte activation (GFAP), synaptic marker changes (PSD95), and NF-κB pathway activation. In vitro, minocycline inhibited IL-1β-induced NF-κB nuclear translocation in astrocytes.	[243]
ROCK Inhibitors	Y27632	Inflammatory (formalin)	DLC2 deficiency enhances inflammatory pain by disinhibiting the RhoA/ROCK signaling pathway in spinal microglia. In formalin-induced pain models, DLC2 knockout mice showed increased mechanical and thermal hypersensitivity accompanied by elevated ROCK1/2, IL-1β, and p38 MAPK activation and increased microglial activation. Pharmacological inhibition of RhoA or ROCK reduced pain behaviors and downregulated pro-inflammatory signaling.	[244]
		Neuropathic (PSNI)	PSNI induces neuropathic pain associated with activation of spinal RhoA/ROCK signaling, increased glial activation (GFAP and Iba-1), and MARCKS phosphorylation. Pharmacological ROCK inhibition (Y27632) blocked RhoA activation, reduced astrogliosis, and prevented behavioral hypersensitivity.	[245]
	Fasudil	Neuropathic (Minamata disease model)	In a Minamata disease rat model, inhibition of the ROCK pathway with fasudil prevented microglial activation in the spinal dorsal horn and blocked maladaptive cortical plasticity.	[246]
COX-2 inhibitors	NS398	Inflammatory (CFA)	Peripheral inflammation induces COX-2 expression in the CNS, driven mainly by IL-1β signaling, leading to increased spinal PGE2 production and central sensitization. This central prostanoid synthesis contributes to mechanical hyperalgesia and widespread inflammatory pain. Inhibition of COX-2 activity within the spinal cord reduces PGE2 levels and alleviates inflammation-induced pain hypersensitivity.	[247]
	Diclofenac NS398	Inflammatory (λ-carrageenan)	Carrageenan-induced peripheral inflammation triggers central sensitization accompanied by COX-2 induction in CNS vascular endothelial cells, leading to increased cerebrospinal PGE2 levels. I.t. COX-2 inhibition reduces both PGE2 production and inflammatory pain hypersensitivity.	[248]
	Indomethacin	Neuropathic (SNL)	Nerve injury induces a transient upregulation of COX-2 in the dorsal spinal cord and thalamus, while COX-1 remains unchanged. This early COX-2 increase is associated with the development of tactile allodynia. I.t. administration of indomethacin prevents the onset of neuropathic pain when administered shortly after injury, but has no effect once pain is established.	[249]

## Data Availability

No new data were created or analyzed in this study. Data sharing is not applicable to this article.

## Abbreviations

The following abbreviations are used in this manuscript:

[^14^C]	Carbon-14
^131^I-BSA	Iodine-131-labeled bovine serum albumin
A1	A1 reactive astrocyte
ABC	ATP-binding cassette
ABCB1	ATP-binding cassette subfamily B member 1
ABCG2	ATP-binding cassette subfamily G member 2
AD	Alzheimer’s Disease
ADP	Adenosine diphosphate
Akt/PKB	Protein kinase B
Ang-1	Angiopoietin 1
Ang-2	Angiopoietin 2
AP-1	Adaptor protein complex 1
AP-2	Adaptor protein complex 2
aPKC	Atypical protein kinase C complex
ApoE3	Apolipoprotein E3
AQP-4	Aquaporin 4
Arp2/3	Actin-related protein 2/3 complex
ASC	Apoptosis-associated speck-like protein containing a CARD
ATP	Adenosine triphosphate
BBB	Blood–brain barrier
BCP	Bone cancer pain
BDNF	Brain-derived neurotrophic factor
BH4	Tetrahydrobiopterin
BMEC	Brain microvascular endothelial cell
C1q	Complement component 1q
Ca^2+^	Calcium ion
CaMKII	Calcium/calmodulin-dependent protein kinase II
Cav-1	Caveolin 1
CCI	Chronic constriction injury
CCL2	C-C motif chemokine ligand 2
CCL5	C-C Motif chemokine ligand 5
CCR2	C-C chemokine receptor type 2
CD11b	Cluster of differentiation 11b (integrin αM)
CD14	Cluster of differentiation 14
CD18	Cluster of differentiation 18 (integrin β2)
CD31	Cluster of differentiation 31 (platelet endothelial cell adhesion molecule-1/PECAM-1)
CD98	Cluster of differentiation 98 (4F2 cell-surface antigen heavy chain)
CD99	Cluster of differentiation 99 (microphage antigen 2/MIC2)
Cdc42	Cell division control protein 42
CFA	Complete Freund’s Adjuvant
CGRP	Calcitonin gene-related peptide
CK2	Casein kinase 2
CLDN5	Claudin 5
CME	Clathrin-mediated endocytosis
CNS	Central nervous system
COX-1	Cyclooxygenase 1
COX-2	Cyclooxygenase 2
CREB	cAMP response element-binding protein
CSF	Cerebrospinal fluid
CX3CL1	C-X3-C motif chemokine ligand 1
CX3CR1	C-X3-C motif chemokine receptor 1
Cx43	Connexin 43
Cx45	Connexin 45
CXCL10	C-X-C motif chemokine ligand 10
CXCL12	C-X-C motif chemokine ligand 12
DAMP	Damage-associated molecular pattern
DHA	Docosahexaenoic acid
Dlg	Drosophila discs large protein
DLC2	Deleted in liver cancer 2
DLL4	Delta-like ligand 4
DNA	Deoxyribonucleic acid
EAAT1	Excitatory amino acid transporter 1
EAAT2	Excitatory amino acid transporter 2
EBA	Evans blue albumin
EGF	Epidermal growth factor
eNOS	Endothelial nitric oxide synthase
ERK1/2	Extracellular signal-regulated kinases 1 and 2
FAK	Focal adhesion kinase
FOXO1	Forkhead box O1
FUS	Focused ultrasound
GABA	Gamma-aminobutyric acid
GABA_A_	Gamma-aminobutyric acid type A receptor
GDNF	Glial cell line-derived neurotrophic factor
GEF-H1	Guanine nucleotide exchange factor-H1
GFAP	Glial fibrillary acidic protein
GLUT1	Glucose transporter type 1
GSH	Reduced glutathione
GSSG	Oxidized glutathione
GTPase	Guanosine triphosphatase
H3K27	Histone H3 lysine 27
HDAC1/2	Histone deacetylases 1 and 2
I.cv.	Intracerebroventricular injection
I.pl.	Intraplantar injection
I.t.	Intrathecal injection
I.v.	Intravenous injection
Iba-1	Ionized calcium-binding adaptor molecule 1
iBEC	Induced blood–brain barrier endothelial cell
ICAM-1	Intercellular adhesion molecule 1
IFN-γ	Interferon gamma
IKK	IκB kinase complex
IKKα	IκB kinase alpha
IKKβ	IκB kinase beta
IL-17	Interleukin 17
IL-18	Interleukin 18
IL-1R1	Interleukin 1 receptor type 1
IL-1β	Interleukin 1 beta
IL-6	Interleukin 6
iNOS	Inducible nitric oxide synthase
IoN	Infraorbital nerve injury
IRAK-4	Interleukin-1 receptor-associated kinase 4
JAK/STAT	Janus kinase/signal transducer and activator of transcription
JAK2	Janus kinase 2
JAM	Junctional adhesion molecule
JAM-A	Junctional adhesion molecule A
JAM-B	Junctional adhesion molecule B
JAM-C	Junctional adhesion molecule C
JNK	c-Jun N-terminal kinase
K^+^	Potassium ion
KCC2	Potassium-chloride cotransporter 2
kDa	Kilodalton
Kir	Inwardly rectifying potassium channel
Kir4.1	Inwardly rectifying potassium channel subfamily J member 10
LAT1	L-type amino acid transporter 1
LDN	Low-dose naltrexone
LFA-1	Lymphocyte function-associated antigen 1
LPC	Lysophosphatidylcholine
LRP5/6	Low-density lipoprotein receptor-related protein 5/6
mAb	Monoclonal antibody
MAPK	Mitogen-activated protein kinase
MARCKS	Myristoylated alanine-rich C-kinase substrate
MCT1	Monocarboxylate transporter 1
MCT2	Monocarboxylate transporter 2
Mfsd2a	Major facilitator superfamily domain-containing protein 2A
MLC	Myosin light chain
MLCP	Myosin light chain phosphatase
MMP-2	Matrix metalloproteinase 2
MMP-9	Matrix metalloproteinase 9
mPGES-1	Microsomal prostaglandin E synthase 1
mRNA	Messenger RNA
MRP	Multidrug resistance-associated protein
MyD88	Myeloid differentiation primary response 88
Na^+^	Ion sodium
NADPH	Nicotinamide adenine dinucleotide phosphate
NEMO	NF-κB essential modulator
NF-κB	Nuclear factor kappa-light-chain-enhancer of activated B cells
NLRP3	NOD-, LRR- and pyrin domain-containing protein 3
NMDA	N-methyl-D-aspartate
NMDAR	N-methyl-D-aspartate receptor
NO	Nitric oxide
NOX2	NADPH oxidase 2
NOX4	NADPH oxidase 4
NR2B	NMDA receptor subunit GluN2B
NRF2	Nuclear factor erythroid 2-related factor 2
NSAID	Non-steroidal anti-inflammatory drug
OCLN	Occludin
ONOO^−^	Peroxynitrite
OX-42	CD11b microglial marker
p115-RhoGEF	p115 Rho guanine nucleotide exchange factor
P2X4	P2X purinergic receptor 4
P2X7	P2X purinergic receptor 7
p38	p38 MAPK
p38 MAPK	p38 mitogen-activated protein kinase
PALS1	Protein associated with lin seven 1
PAR	Partitioning-defective
PATJ	PALS1-associated tight junction protein
PDGF-B	Platelet-derived growth factor B
PDZ	PSD-95/Dlg/ZO-1 domain
PECAM-1	Platelet/endothelial cell adhesion molecule 1 (also CD31)
PI3K/Akt	Phosphoinositide 3-kinase
PGE2	Prostaglandin E2
PHN	Postherpetic neuralgia
PIP_2_	Phosphatidylinositol 4,5-bisphosphate
PKA	Protein kinase A
PKC	Protein kinase C
PKCζ	Protein kinase C zeta
PNI	Peripheral nerve injury
PPARγ	Peroxisome proliferator-activated receptor gamma
PRR	Pattern recognition receptor
PSD-95	Postsynaptic density protein 95
PSNL	Partial sciatic nerve ligation
PTX	Paclitaxel
Rab5	Ras-related in brain protein 5
Rac1	Ras-related C3 botulinum toxin substrate 1
Rap1	Ras-related protein 1
Rho	Ras homologous GTPase
Rho GTPase	Ras homolog family GTPase
RhoA	Ras homolog family member A
RIPK1	Receptor-interacting serine/threonine-protein kinase 1
ROCK	Rho-associated protein kinase
ROCK1/2	Rho-associated coiled-coil containing protein kinase 1 and 2
ROCK1	Rho-associated coiled-coil containing protein kinase 1
ROCK2	Rho-associated coiled-coil containing protein kinase 2
ROS	Reactive oxygen species
s.c.	Subcutaneous injection
S1PR1	Sphingosine-1-phosphate receptor 1
SCI	Spinal cord injury
SH3	Src homology 3 domain
SLC2A1	Solute carrier family 2 member 1
SMAD	Sma and Mad-related proteins
SNL	Spinal nerve ligation
SOD	Superoxide dismutase
Src	Sarcoma kinase family
STAT3	Signal transducer and activator of transcription 3
STZ	Streptozotocin
TBARS	Thiobarbituric acid reactive substances
TEER	Transendothelial electrical resistance
TGF-β	Transforming growth factor beta
Tie2	TEK receptor tyrosine kinase
TIMP	Tissue inhibitor of metalloproteinases
TIMP-1	Tissue inhibitor of metalloproteinases 1
TIMP-2	Tissue inhibitor of metalloproteinases 2
TJP1	Tight junction protein 1
TLR4	Toll-like receptor 4
TNC	Trigeminal nucleus caudalis
TNFR1	Tumor necrosis factor receptor 1
TNF-α	Tumor necrosis factor alpha
TRADD	TNFR1-associated death domain protein
TRAF2	TNF receptor-associated factor 2
TRAF6	TNF receptor-associated factor 6
TRIF	TIR-domain-containing adapter-inducing interferon-β
Tyr	Tyrosine
VCAM-1	Vascular cell adhesion molecule 1
VE-cadherin	Vascular endothelial cadherin
VEGF	Vascular endothelial growth factor
VEGF-A	Vascular endothelial growth factor A
VEGFR2	Vascular endothelial growth factor receptor 2
VLA-4	Very late antigen 4
WASP	Wiskott–Aldrich syndrome protein
WAVE	WASP-family verprolin homologous protein complex
Wnt	Wingless/integrated
Wnt7a	Wnt family ligand 7A
Wnt7b	Wnt family ligand 7B
ZO	Zonula occludens
ZO-1	Zonula occludens 1
ZO-2	Zonula occludens 2
ZO-3	Zonula occludens 3
ZONAB	Zonula occludens-1 associated nucleic acid-binding protein
ω-3	Omega-3
